# Conservation and expansion of a necrosis‐inducing small secreted protein family from host‐variable phytopathogens of the Sclerotiniaceae

**DOI:** 10.1111/mpp.12913

**Published:** 2020-02-15

**Authors:** Matthew Denton‐Giles, Hannah McCarthy, Tina Sehrish, Yasmin Dijkwel, Carl H. Mesarich, Rosie E. Bradshaw, Murray P. Cox, Paul P. Dijkwel

**Affiliations:** ^1^ Centre for Crop and Disease Management Curtin University Perth Australia; ^2^ School of Fundamental Sciences Massey University Palmerston North New Zealand; ^3^ School of Agriculture and Environment Massey University Palmerston North New Zealand

**Keywords:** *Botrytis cinerea*, *Ciborinia camelliae*, gene knockout, necrosis‐inducing proteins, recombinant protein expression, *Sclerotinia sclerotiorum*, small secreted proteins

## Abstract

Fungal effector proteins facilitate host‐plant colonization and have generally been characterized as small secreted proteins (SSPs). We classified and functionally tested SSPs from the secretomes of three closely related necrotrophic phytopathogens: *Ciborinia camelliae*, *Botrytis cinerea,* and *Sclerotinia sclerotiorum*. Alignment of predicted SSPs identified a large protein family that share greater than 41% amino acid identity and that have key characteristics of previously described microbe‐associated molecular patterns (MAMPs). Strikingly, 73 of the 75 SSP family members were predicted within the secretome of the host‐specialist *C. camelliae* with single‐copy homologs identified in the secretomes of the host generalists *S. sclerotiorum* and *B. cinerea*. To explore the potential function of this family of SSPs, 10 of the 73 *C. camelliae* proteins, together with the single‐copy homologs from *S. sclerotiorum* (SsSSP3) and *B. cinerea* (BcSSP2), were cloned and expressed as recombinant proteins. Infiltration of SsSSP3 and BcSSP2 into host tissue induced rapid necrosis. In contrast, only one of the 10 tested *C. camelliae* SSPs was able to induce a limited amount of necrosis. Analysis of chimeric proteins consisting of domains from both a necrosis‐inducing and a non‐necrosis‐inducing SSP demonstrated that the C‐terminus of the *S. sclerotiorum* SSP is essential for necrosis‐inducing function. Deletion of the *BcSSP2* homolog from *B. cinerea* did not affect growth or pathogenesis. Thus, this research uncovered a family of highly conserved SSPs present in diverse ascomycetes that exhibit contrasting necrosis‐inducing functions.

## Graphical abstract

The C‐terminus of previously uncharacterized small, secreted, cysteine‐rich proteins from three fungal pathogens of the Sclerotiniaceae is responsible for necrosis‐inducing function in plant tissues.

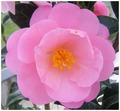

## 1 INTRODUCTION

Some of the most economically important eukaryotic phytopathogens are fungi (Dean *et al.*, 2012). Confined mainly to the Ascomycota and Basidiomycota, these fungi have evolved the means to penetrate plant tissue and sequester valuable nutrients, all at great expense to the host plant. The lifestyles of fungal phytopathogens vary, from obligate biotrophs that are unable to survive outside host tissue, to broad‐host necrotrophs that sequester nutrients from necrotized tissue (Oliver and Ipcho, 2004).

Independent of their lifestyles, all phytopathogenic fungi secrete virulence factors, also known as effectors, to aid in the establishment and development of infection within their host(s) (Cook *et al.*, 2015; Lo Presti *et al.*, 2015). Fungal effectors consist of a diverse group of molecules, including toxic secondary metabolites, enzymatic proteins, nonenzymatic proteins, and small interfering RNA molecules (Howlett, 2006; Stergiopoulos and de Wit, 2009; Weiberg *et al.*, 2013; Collemare *et al.*, 2019). Many fungal effectors discovered previously are small, cysteine‐rich proteins that are secreted during host infection (Stergiopoulos and de Wit, 2009).

The mechanisms by which proteinaceous effectors influence the host are extremely diverse and have been well described for several biotrophic fungi. The *Cladosporium fulvum* Avr2 effector actively suppresses the host immune system by inhibiting host proteases that normally function to degrade fungal peptides in the host apoplast (Rooney *et al.*, 2005; van Esse *et al.*, 2008). In a less direct manner, the *C*. *fulvum* Avr4 effector suppresses plant chitinase activity by forming a protective coat of protein over the fungal cell wall, preventing chitinases from binding and degrading fungal chitin (van Esse *et al.*, 2007). Ecp6 of *C*. *fulvum* scavenges chitin oligosaccharides in order to prevent the elicitation of the host immune system by these molecules (de Jonge *et al.*, 2010). The *Ustilago maydis* chorismate mutase effector uses its enzymatic activity to coordinate changes to the biosynthesis of antifungal compounds in cells proximal to the infection zone (Djamei *et al.*, 2011).

More recently, proteinaceous effectors of necrotrophic pathogens have also been described (Tan *et al.*, 2010). The majority act to promote host‐cell death in accordance with the lifestyles of these plant pathogens (Friesen *et al.*, 2007; Lorang *et al.*, 2012). Wheat pathogens *Parastagonospora nodorum* and *Pyrenophora tritici*‐*repentis* both secrete the proteinaceous effector ToxA during host infection (Friesen *et al.*, 2006). ToxA has been shown to interact indirectly with the host's Tsn1 protein to facilitate host‐cell death and susceptibility (Faris *et al.*, 2010). Only host genotypes that contain the *Tsn1* “sensitivity” gene are susceptible to ToxA‐mediated cell death. Additional Tox proteins of *P*. *nodorum* have also been shown to act in conjunction with sensitivity proteins, including SnTox1, SnTox2, SnTox3, and SnTox4 (Liu *et al.*, 2009, 2012).

Traditionally, proteinaceous fungal effectors were identified by their ability to trigger a hypersensitive response in incompatible host tissue (Lauge and De Wit, 1998). More recently, it has become possible to predict putative fungal effectors using bioinformatic analyses. In particular, fungal secretome prediction has become a popular strategy to identify proteinaceous fungal effectors (Amselem *et al.*, 2011; Hacquard *et al.*, 2012; Morais do Amaral *et al.*, 2012; Guyon *et al.*, 2014; Heard *et al.*, 2015; Derbyshire *et al.*, 2017). Secreted fungal proteins contain N‐terminal signal peptides that guide these proteins through the classical secretion pathway (Lippincott‐Schwartz *et al.*, 2000). Together with transmembrane domain prediction tools, signal peptide sequence prediction analyses have been used to identify fungal secretomes from fungal proteomes (Emanuelsson *et al.*, 2000; Petersen *et al.*, 2011). The identification of putative fungal effectors within a secretome has traditionally involved filtering for small proteins (<200 amino acids) with a high cysteine content (Templeton *et al.*, 1994; Hacquard *et al.*, 2012). The cysteine residues within fungal effector proteins are proposed to form disulphide bonds, which help maintain protein stability within plant tissue (Joosten *et al.*, 1997; Luderer *et al.*, 2002). More recently, effector screening strategies have begun to incorporate complex information, including temporal and tissue‐specific gene expression patterns, evidence for positive selection, proteomics, three‐dimensional protein structure prediction, and comparative secretome analyses (Pedersen *et al.*, 2012; Guyon *et al.*, 2014; de Guillen *et al.*, 2015; Lo Presti *et al.*, 2015; Sperschneider *et al.*, 2015; Heard *et al.*, 2015; Mesarich *et al.*, 2018).

Proteinaceous fungal effectors are often under strong selection pressure and must constantly evolve at the molecular level to maintain their function (Rouxel *et al.*, 2011; Sperschneider *et al.*, 2014). However, some groups of proteinaceous effector molecules are homologous at the protein sequence level. Previously characterized homologous fungal effectors belong to the necrosis and ethylene‐inducing peptide 1 (NEP1)‐like protein (NLP) family, the cerato‐platanin protein family, and the homologs of *C. fulvum* Ecp2 (Hce2) family (Stergiopoulos *et al.*, 2012; Santhanam *et al.*, 2013; Gaderer *et al.*, 2014). Notably, these effectors tend to function as host‐cell death inducers, as opposed to plant immune system suppressors (Bailey, 1995; Staats *et al.*, 2007; Dallal *et al.*, 2010; Stergiopoulos *et al.*, 2010; Frías *et al.*, 2011; Oome *et al.*, 2014). Therefore, by screening fungal secretomes for conserved small secreted proteins (SSPs), it may be possible to select for proteins that induce host‐cell necrosis.

To test this hypothesis, we predicted and compared the secretomes of the three necrotrophic fungal phytopathogens *Botrytis cinerea*, *Sclerotinia sclerotiorum*, and *Ciborinia camelliae*. All three of these fungal species are closely related members of the Sclerotiniaceae, sharing the ability to produce sexual fruiting bodies (apothecia) from melanized masses of mycelia (sclerotia) (Whetzel, 1945). Despite their similar necrotrophic lifestyles and taxonomic classification, the number of hosts that each of these pathogens infects varies considerably. It is estimated that *B. cinerea* and *S. sclerotiorum* have >1,400 and >400 host species, respectively, including the important crop species *Glycine max* (soybean), *Brassica napus* (canola), and *Vitis vinifera* (grape) (Boland and Hall, 1994; Bolton *et al.*, 2006; van Kan *et al.*, 2017). In contrast, the host range of *C. camelliae* is restricted solely to the floral organs of some *Camellia* species and interspecific hybrids (Kohn and Nagasawa, 1984; Denton‐Giles *et al.*, 2013). Here, we describe a bioinformatic approach that resulted in the discovery of a new family of conserved SSPs in *B. cinerea*, *S. sclerotiorum*, and *C. camelliae* that were massively expanded in the latter restricted host‐range pathogen. We investigated the evolution of these novel SSPs within host‐variable pathogens, their putative functions, and their role in fungal virulence.

## 2 RESULTS

### 2.1 Comparative analysis of the *C. camelliae*, *B. cinerea*, and *S. sclerotiorum* secretomes reveals an enrichment of cysteine‐rich SSPs in *C. camelliae*

A total of 14,711 nonredundant *C. camelliae* protein sequences were predicted from genomic and transcriptomic data. Secretome prediction was performed for all three fungal species as outlined in Figure S1. Protein sequences were screened for the presence of signal peptides, cellular localization signals, and the absence of transmembrane domains. A total of 749 *C. camelliae*, 754 *B. cinerea*, and 677 *S. sclerotiorum* secreted protein sequences were predicted.

**Figure 1:**
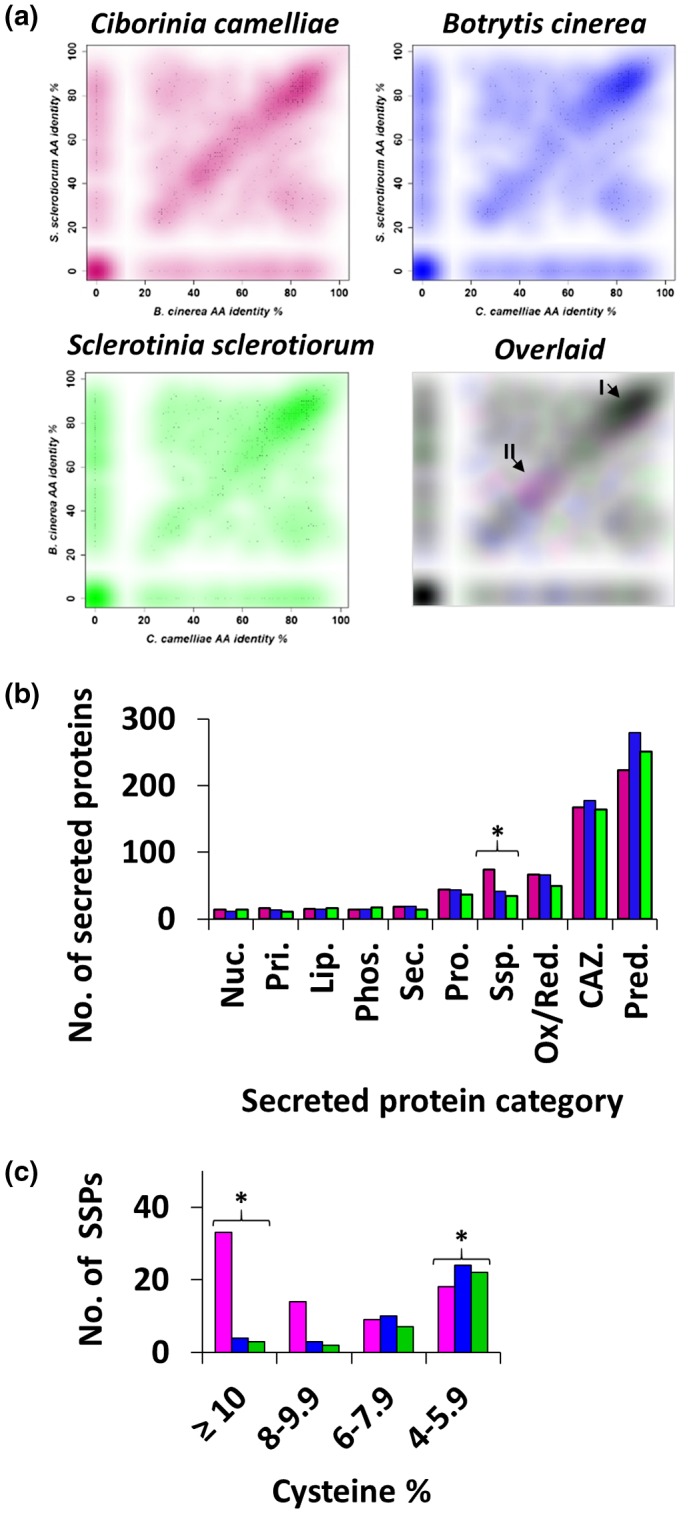
Prediction and comparative analyses of the secretomes of *Ciborinia camelliae* (pink), *Botyrtis cinerea* (blue), and *Sclerotinia sclerotiorum* (green). (a) Scatterplot analysis of the secretomes of *C. camelliae*, *B. cinerea*, and *S. sclerotiorum*. Predicted secreted proteins from each fungal pathogen were aligned to predicted secreted proteins from the other two fungal pathogens using BLASTP. Each query sequence produced two “best hit” amino acid (AA) identity scores. Three graphs were independently generated and were overlaid for comparison. I, a cluster of highly conserved proteins; II, a dominant cluster of *C*. *camelliae*‐specific proteins. (b) A comparison of the annotated fungal secretomes of *C*. *camelliae*, *B*. *cinerea*, and *S*. *sclerotiorum*. Raw counts represent the number of proteins in each gene ontology category. The top 10 most common categories are shown. Nuc., nucleic acid modification proteins; Pri., primary metabolism proteins; Lip., lipases; Phos., phosphatases; Sec., secondary metabolism proteins; Pro., proteases; SSP., small secreted proteins; Ox/Red., oxidoreductases; CAZ., carbohydrate‐active enzymes; Pred., predicted proteins. (c) A histogram displaying the distribution of SSPs for each species based on their cysteine content. Asterisks indicate statistical differences (Fisher's exact test using a 3 × 2 contingency table) (*p* < .001)

To determine the level of conservation between the secretomes of *S. sclerotiorum*, *B. cinerea*, and *C. camelliae*, individual proteins from each species were independently aligned to the secretomes of the other two species. The single best alignment to each species was identified and amino acid identity information was plotted on a two‐dimensional scatterplot, producing a spatial representation of secreted protein conservation (Figure 1a). Each of the three scatter plots produced a discernible positive slope from the lower left of the plot to the upper right. Secreted proteins that produced comparable amino acid identity scores when aligned to the other two secretomes mapped along the positive slope. The strongest cluster of proteins formed in the top right of each scatter plot, suggesting that a large proportion of the secreted proteins shared high amino acid identity across all three fungal species (Figure 1a, arrow I). A second densely coloured cluster routinely appeared at the *x*, *y* coordinates 0, 0. Proteins at this position had no discernible homology to the other secretomes and are likely to be either species‐specific or incorrectly predicted. Overlaying the three scatterplots identified a region of divergence where proteins from a single species dominated (Figure 1a, arrow II).

To determine which types of proteins were conserved or divergent, all the secreted proteins were annotated using BLAST2GO (Conesa and Götz, 2008). A total of 76%–80% of proteins within each secretome were assigned to gene ontology (GO) categories of predicted protein (30%–35%), CAZyme (20%–25%), oxidoreductase (7%–10%), SSP (5%–10%) or protease (5%) (Figure 1b). The remaining proteins were distributed among 27 additional categories (Figure 1b and Table S1).

The most striking difference between the secretomes of all three species appeared within the SSP category. *C. camelliae* had substantially more SSPs compared to *B. cinerea* and *S. sclerotiorum* (Fisher's exact test, *p* < .001) (Figure 1b). SSPs with ≥8% cysteine composition were more abundant within the *C. camelliae* secretome (*p* < .001) (Figure 1c) and were the dominant group contributing to the *C. camelliae*‐specific cluster identified in the overlaid scatter plot (Figure 1a, arrow II). Significantly more SSPs with 4.0%–5.9% cysteine content were present in the *B. cinerea* and *S. sclerotiorum* secretomes as compared to the *C.* *camelliae* secretome (*p* < .001) (Figure 1c).

### 2.2 A family of cysteine‐rich SSPs are conserved across the secretomes of *C. camelliae*, *B. cinerea*, and *S. sclerotiorum*

**Figure 2:**
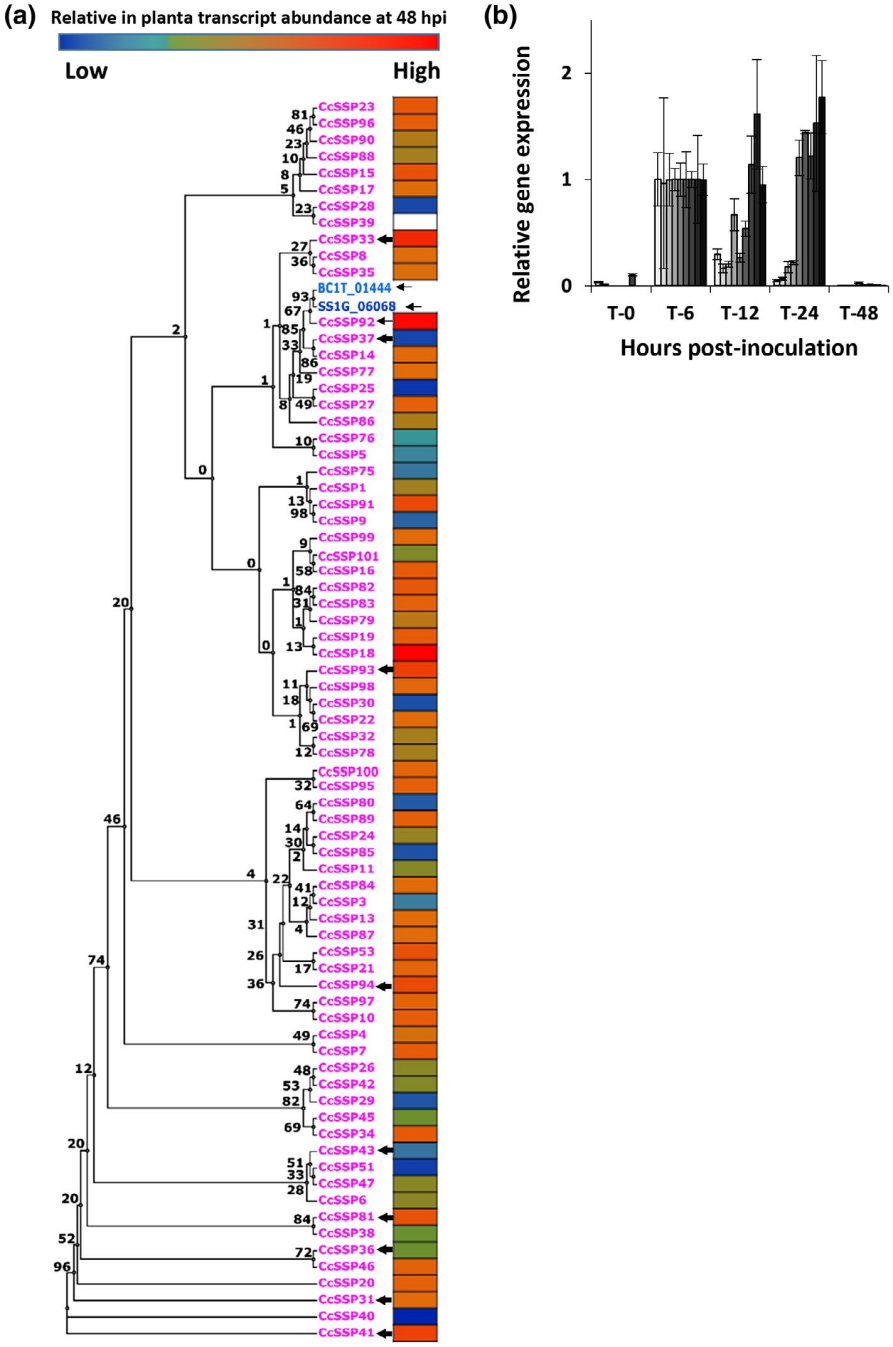
(a) Phylogenetic analysis of the conserved small secreted protein (SSP) family. Relative in planta transcript abundance was calculated for each of the conserved *Ciborinia camelliae* SSPs. Arrows indicate the 12 SSPs that were cloned and expressed as recombinant proteins and include those that were chosen for quantitative reverse transcription PCR (RT‐qPCR) analysis (thick arrows). (b) RT‐qPCR data for a subset of nine of the 73 *C. camelliae SSP* genes. All data were normalized to the two fungal housekeeping genes *NAD* and *TUB*. Histogram bars from lightest to darkest represent the expression of *CcSSP37, CcSSP94, CcSSP36, CcSSP31, CcSSP93, CcSSP33, CcSSP41, CcSSP43*, and *CcSSP81*. Relative expression data were normalized to 6 hr post‐inoculation to allow for comparisons between genes. Error bars = ±1 *SD*

To investigate the level of conservation between the SSPs of *S. sclerotiorum*, *B. cinerea*, and *C. camelliae*, a cladogram was built from an alignment of all 148 SSPs (Figure S2). A highly supported clade of 48 conserved proteins emerged from the cladogram, with 46 proteins belonging to *C. camelliae*, 1 to *B. cinerea*, and 1 to *S. sclerotiorum*. The *C. camelliae* members were confirmed as the cysteine‐rich (>8%) SSPs that contribute to the *C. camelliae*‐dominant cluster (Figure 1a, arrow II). Mining the *C. camelliae* genome for homologs of this protein family identified an additional 26 proteins. These additional proteins had not been predicted in the secretome due to their shorter length, absence of an N‐terminal methionine, or other unknown factors. Equivalent analyses in the *B. cinerea* and *S. sclerotiorum* genomes confirmed that only single‐copy homologs of this conserved gene family existed within these two species. Analysis of the spatial positioning of the *C. camelliae* genes across the scaffolds of the *C. camelliae* draft genome indicated that they are clustered (Figure S3). In total, 73 unique coding sequences belonging to this protein family were identified in *C. camelliae*, 1 in *B. cinerea* (*BC1T_01444*), and 1 in *S. sclerotiorum* (*SS1G_06068*) (Figure 2a and Table S3). Collectively these proteins will be henceforth referred to as *Ciborinia camelliae*‐like small secreted proteins (CCL‐SSPs).

All 75 CCL‐SSP family members were predicted to contain an N‐terminal signal peptide (Table S3). The predicted signal cleavage site was followed by a domain containing five conserved cysteine residues, which includes the conserved amino acid motif CTYCQCLFPDGSHCC. All 10 of the cysteine residues were predicted to form disulphide bonds and predicted disulphide connectivity patterns were conserved for all 75 proteins (Table S3). The conservation of cysteine residues suggests that the CCL‐SSPs are likely to maintain a robust secondary structure.

### 2.3 CCL‐SSP homologs are present across fungal classes

To search for additional cross‐species homologs, all 75 of the CCL‐SSPs were aligned to the nonredundant protein database using BLASTP. A total of 23 additional fungal species were identified as having at least one CCL‐SSP homolog (Table 1). All identified species belonged within one of four taxonomical classes: the Leotiomycetes, Dothideomycetes, Eurotiomycetes, and Sordariomycetes. Comparatively, *C. camelliae* had by far the largest number of CCL‐SSP family members (*n* = 73). Pairwise amino acid identity was assessed for each homolog by comparison with CcSSP92, which is the most ancestral *C. camelliae* CCL‐SSP sequence based on its amino acid sequence conservation with BcSSP2 and SsSSP3 (Figure 2a). Interestingly, the CcSSP92 sequence has higher amino acid identity with CCL‐SSP homologues in *B. cinerea*, *S. sclerotiorum*, *Aspergillus flavus*, *S. borealis*, *A. niger*, *A. kawachii*, and *Diaporthe ampelina* than it does with another *C. camelliae* CCL‐SSPs (Table 1). An alignment of all known homologs (*n* = 113) indicated that cysteine residues located from cysteine positions 2 to 8 were highly conserved (≥95%) within the greater CCL‐SSP family (Figure S4).

**Table 1:**
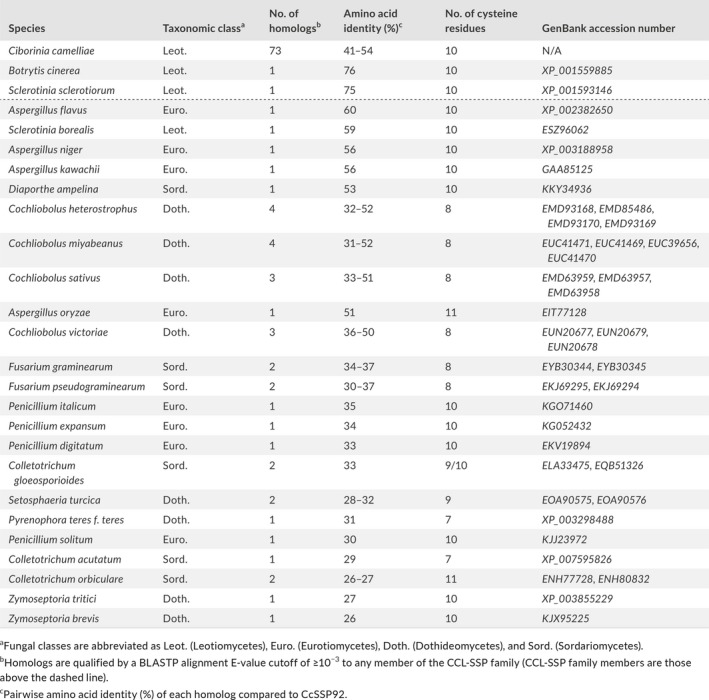
Identification of *C. camelliae*‐like SSPs in other fungi

^a^Fungal classes are abbreviated as Leot. (Leotiomycetes), Euro. (Eurotiomycetes), Doth. (Dothideomycetes), and Sord. (Sordariomycetes).

^b^Homologs are qualified by a BLASTP alignment E‐value cutoff of ≥10^−3^ to any member of the CCL‐SSP family (CCL‐SSP family members are those above the dashed line).

^c^Pairwise amino acid identity (%) of each homolog compared to CcSSP92.

### 2.4 *C. camelliae CCL‐SSPs* are expressed during early infection

To determine whether the expanded family of *CCL‐SSP* genes in *C. camelliae* are actively transcribed during infection, *C. camelliae* in planta RNA‐Seq data were analysed. Transcripts of 72 of the 73 *C. camelliae CCL‐SSP* genes were detected in the *C. camelliae* in planta reference transcriptome (Figure 2a). Transcripts of the *B. cinerea* single‐copy *CCL‐SSP* gene have previously been detected during the onset of disease in *Lactuca sativa* (lettuce) (Cremer *et al.*, 2013). Expression of the *S. sclerotiorum* single‐copy *CCL‐SSP* gene has been detected during infection of *Helianthus annuus* (sunflower) (Guyon *et al.*, 2014) and has been shown to be up‐regulated in hyphal tips during infection of *Arabidopsis thaliana* (Peyraud *et al.*, 2019).

To further characterize the expression of the *C. camelliae CCL‐SSP* genes, temporal expression analysis was performed on nine randomly chosen genes using quantitative reverse transcription PCR (RT‐qPCR) (Figure 2b). Immediately post‐inoculation (0 hr), gene expression was low or undetectable for all nine genes. Gene expression increased for all *CCL‐SSP* homologs by 6 hr post‐inoculation (hpi), which coincides with ascospore germination and cuticle penetration (Denton‐Giles *et al.*, 2013). From 6 to 24 hpi, the expression levels of individual genes varied, with some consistently increasing, some remaining constant, and others declining in expression. Of significance was the decline in expression of all genes by 48 hpi, which coincides with lesion maturation and the onset of host‐cell necrosis (Denton‐Giles *et al.*, 2013). These data demonstrate that this subset of nine *C. camelliae CCL‐SSP*s are more highly expressed during the symptomless prelesion period of in planta development.

### 2.5 Recombinant CCL‐SSP family members induce host‐cell necrosis

**Figure 3:**
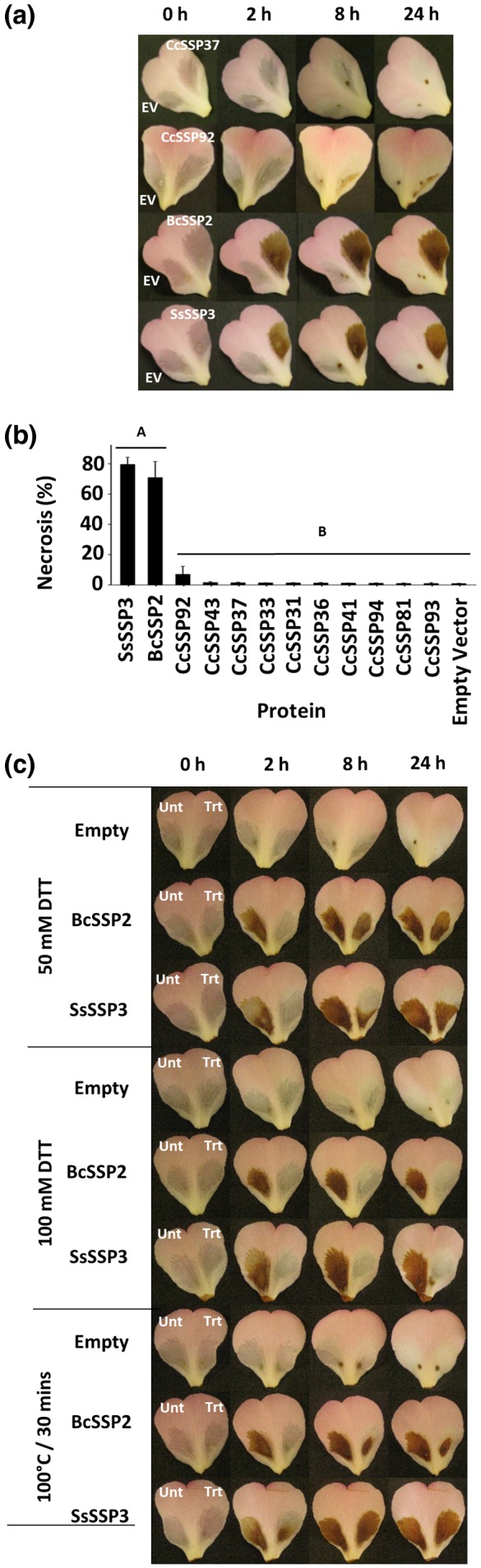
Functional characterization of native recombinant CCL‐SSPs. (a) Culture filtrates derived from *Pichia pastoris* strains containing recombinant BcSSP2, SsSSP3, CcSSP92, and CcSSP37 were infiltrated into *Camellia* 'Nicky Crisp' petal tissue (right petal lobe). Empty vector (“EV”) culture filtrate was co‐infiltrated into the same petals to serve as a negative control (left petal lobe). Representative images are shown (*n* = 3). Photographs were taken at 0, 2, 8, and 24 hr post‐infiltration. (b) Quantitative analysis of the area of necrosis caused by the infiltration of each protein culture filtrate. *p* ≤ .05 (Tukey's HSD), error bars = ±1 *SD*. (c) Culture filtrates derived from *P. pastoris* strains containing recombinant BcSSP2, SsSSP3 or empty vector were treated (Trt) for 2 hr with either 50 mM dithiothreitol (DTT) or 100 mM DTT, or boiling for 30 min. Untreated (Unt) culture filtrate (left petal lobe) or treated culture filtrate (right petal lobe) was infiltrated and photographs were taken at 0, 2, 8, and 24 hr post‐infiltration. Representative images are shown (*n* = 3)

To gain insights into the cellular function of the CCL‐SSP family, 12 *CCL‐SSP* genes (*CcSSP31*, *CcSSP33*, *CcSSP36*, *CcSSP37*, *CcSSP41*, *CcSSP43*, *CcSSP81*, *CcSSP92*, *CcSSP93*, *CcSSP94*, *BcSSP2*, and *SsSSP3*) were selected for cloning and recombinant protein expression in *Pichia pastoris*. The 10 *C. camelliae CCL‐SSP* genes are spread across the phylogenetic spectrum of the *C. camelliae* CCL‐SSP family and include the two homologs that share the greatest amino acid sequence conservation with BcSSP2 and SsSSP3 (Figure 2a). Filter‐sterilized culture filtrates were collected for each recombinant protein and infiltrated into host petal tissue. Nine of the 10 culture filtrates that contained *C. camelliae* CCL‐SSP protein homologs failed to stimulate a visible host response by 24 hr post‐infiltration (Figures 3a and S5a). Culture filtrate harbouring recombinant CcSSP92 protein produced a small area of necrosis from 8 hr post‐infiltration (Figure 3a). However, quantification of CcSSP92‐induced necrosis indicated that it was not significantly higher than the level of necrosis caused by the other nine *C. camelliae* CCL‐SSP proteins (Figure 3b). In contrast, culture filtrates with recombinant BcSSP2 and SsSSP3 proteins stimulated rapid host‐cell necrosis, specific to the infiltrated area, from 2 hr post‐infiltration (Figure 3a,b). BcSSP2 and SsSSP3 culture filtrates also induced a host‐cell necrosis response in *Nicotiana benthamiana*, suggesting that the activity of these two proteins is not host‐specific (Figure S5b).

To confirm that BcSSP2 and SsSSP3 proteins were responsible for the observed host‐cell necrosis phenotype, samples of culture filtrate were collected following induction with buffered methanol complex medium (BMMY) protein induction medium. Culture filtrates collected ≥3 hr post‐induction stimulated host‐cell necrosis (Figure S6). Only culture filtrates that were collected at 0 hr post‐induction failed to stimulate a response, suggesting that the active component of each culture filtrate was accumulated under inductive conditions.

To disrupt the conformation of BcSSP2 and SsSSP3, culture filtrates were treated for 2 hr prior to infiltration with either 50 mM dithiothreitol (DTT) or 100 mM DTT, or were boiled for 30 min. Initially, culture filtrates containing 50 mM DTT were unable to induce host‐cell necrosis. However, by 8 hr post‐infiltration host‐cell necrosis had developed in host‐tissue treated with BcSSP2‐ and SsSSP3‐containing culture filtrates (Figure 3c). Increasing the DTT concentration to 100 mM resulted in a near total loss of the host‐cell necrosis phenotype, although a small lesion was observed in SsSSP3‐treated tissue at 24 hpi. Boiling the BcSSP2 and SsSSP3 culture filtrates for 30 min initially reduced the level of host‐necrosis compared to the unboiled control. However, by 8 hr post‐infiltration the necrosis‐inducing phenotype had appeared. Together these results suggest that the necrosis‐inducing component of the BcSSP2 and SsSSP3 culture filtrates is likely to be a heat‐stable protein.

### 2.6 C‐terminus‐tagged CCL‐SSPs have reduced necrosis‐inducing function

**Figure 4:**
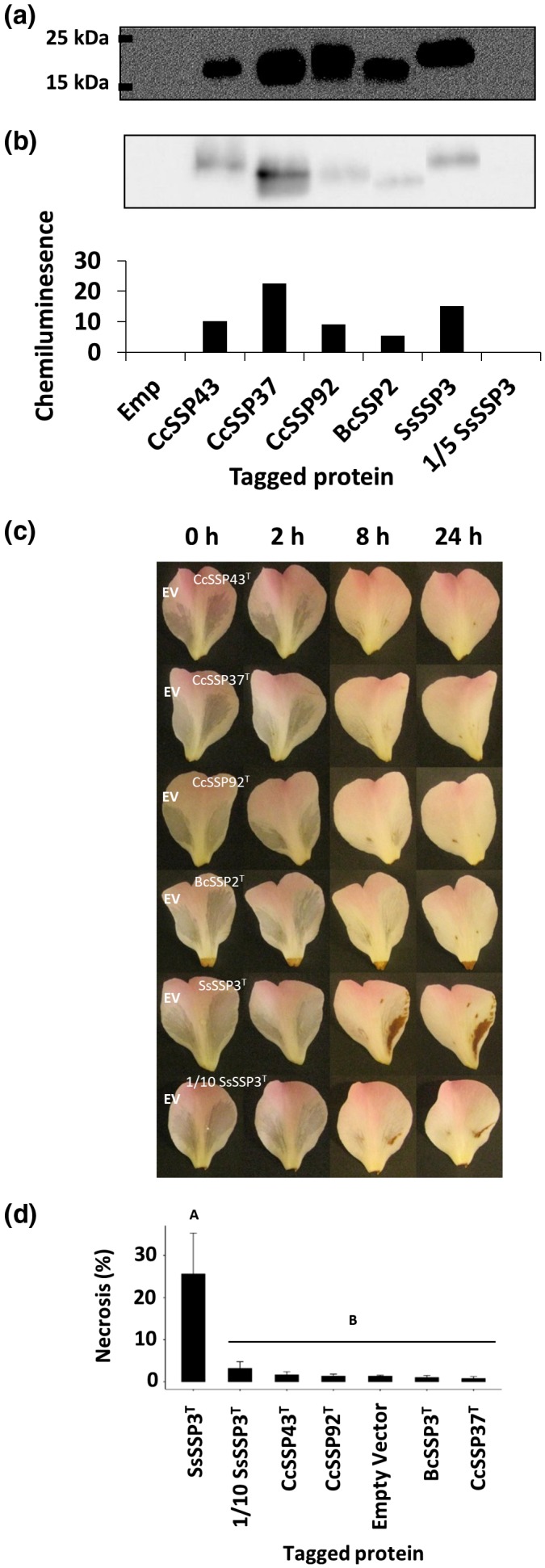
Tagged recombinant CCL‐SSP expression and functional assays. (a) A western blot detecting the expression of five tagged (^T^) CCL‐SSPs. Lane 1, empty vector; lane 2, CcSSP43^T^; lane 3, CcSSP37^T^; lane 4, CcSSP92^T^; lane 5, BcSSP2^T^; lane 6, SsSSP3^T^; lane 7, 1/5 diluted SsSSP3^T^. (b) Semiquantitative analysis of protein abundance. (c) *Camellia* 'Nicky Crisp' petal tissue infiltrated with empty vector (“EV”) culture filtrate (left petal lobe) and culture filtrates containing tagged recombinant proteins (right petal lobe). SsSSP3^T^ was diluted (1/10) to detect the lower bounds of protein concentration required to induce necrosis. Photographs were taken at 0, 2, 8, and 24 hr post‐infiltration. Representative images are shown (*n* = 3). (d) Quantitative analysis of the area of necrosis caused by the infiltration of each tagged protein culture filtrate. *p* ≤ .05 (Tukey's HSD), error bars = ±1 *SD*

The native recombinant proteins BcSSP2 and SsSSP3 induce strong host‐cell necrosis. However, it is unclear whether the lack of necrosis from the native *C. camelliae* CCL‐SSP proteins was due to a lack of synonymous function or reduced concentrations of soluble protein. To facilitate the determination of CCL‐SSP protein concentration, a c‐Myc 6 × His‐tag was included at the C‐terminus of CcSSP43^T^, CcSSP37^T^, CcSSP92^T^, BcSSP2^T^, and SsSSP3^T^. The presence of tagged CCL‐SSP proteins in culture filtrate was confirmed by western blot using antibodies raised against the c‐Myc tag (Figure 4a). To normalize for variations in protein concentration, the concentration of each tagged protein was semiquantified using a chemiluminescence‐based quantification method. All tagged proteins were present in culture filtrates at a higher concentration than the 5‐fold diluted SsSSP3^T^ protein (Figure 4b). Only SsSSP3^T^ undiluted and diluted (10‐fold) culture filtrates were able to induce a host‐cell necrosis response (Figure 4c,d). Compared to the native protein assays, the SsSSP3^T^ host‐necrosis phenotype was delayed in its response and never completely necrotized the infiltrated area. BcSSP3^T^ also failed to induce any visible host‐cell necrosis response, suggesting that the addition of the c‐Myc 6 × His‐tag to the C‐terminus of the native BcSSP3 and SsSSP3 proteins perturbs necrosis‐inducing function.

### 2.7 The C‐terminal region of SsSSP3 is essential for necrosis‐inducing activity

**Figure 5:**
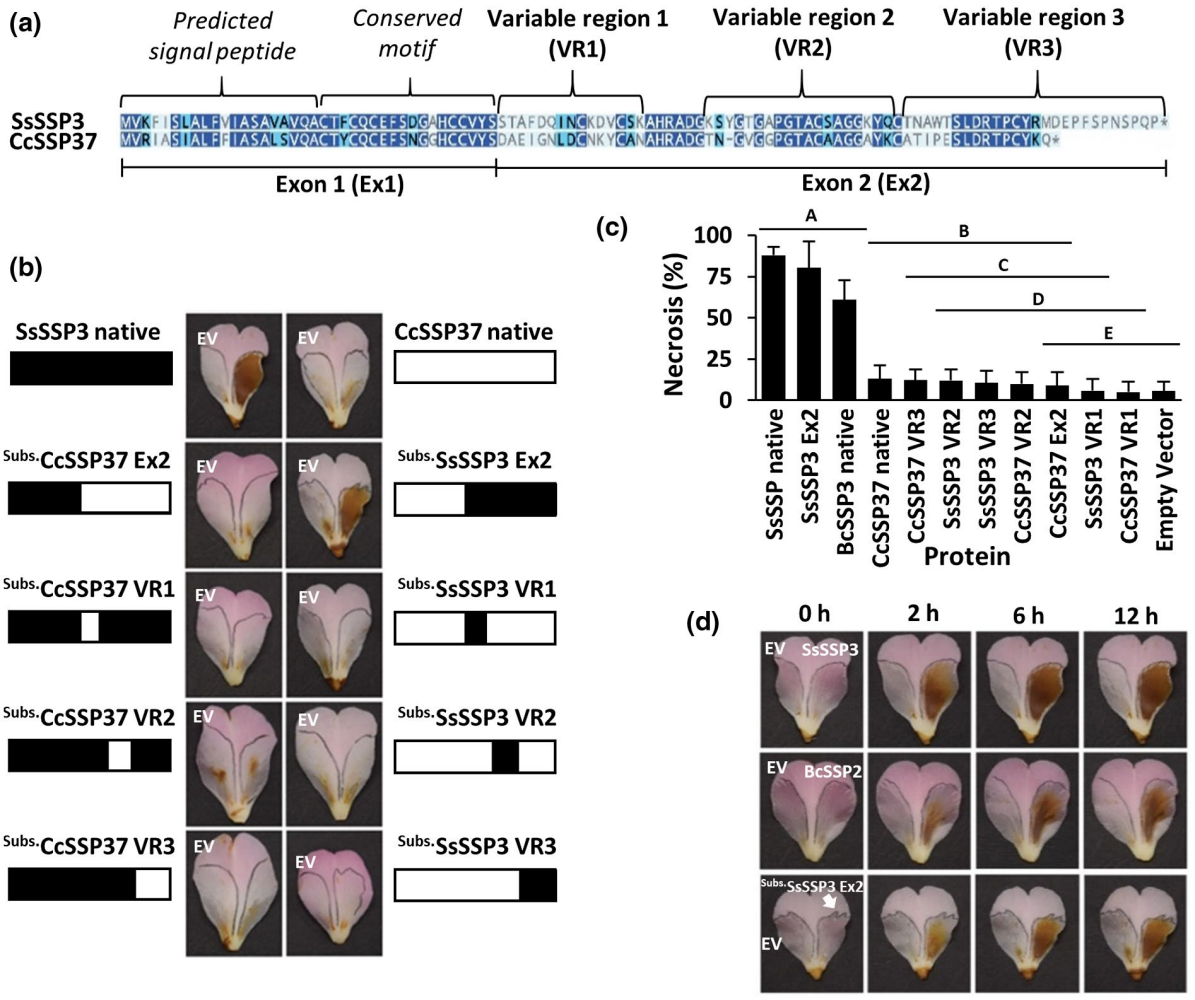
Characterization of chimeric SsSSP3 and CcSSP37 recombinant proteins. (a) Pairwise alignment of SsSSP3 and CcSSP37 highlighting the domains chosen for chimeric protein development. (b) Culture filtrates derived from *Pichia pastoris* strains containing recombinant SsSSP3, CcSSP37, and SsSSP3/CcSSP37 chimeric proteins were infiltrated into *Camellia* 'Nicky Crisp' petal tissue (right petal lobe). Empty vector (“EV”) culture filtrate was co‐infiltrated into the same petals to serve as a negative control (left petal lobe) (*n* = 9). (c) Quantitative analysis of the area of necrosis caused by the infiltration of each protein culture filtrate. *p* ≤ .05 (Tukey's HSD), error bars = ±1 *SD*. (d) Temporal development of necrosis within petal tissue infiltrated with culture filtrate containing SsSSP3, BcSSP2, and ^Subs.^SsSSP3 Ex2 (right lobe), or EV culture filtrate (left lobe) at 0, 2, 6, and 12 hr post‐infiltration

To determine which regions of the CCL‐SSPs contribute to the necrosis‐inducing phenotype, chimeric proteins were created by swapping domains of the native necrosis‐inducing SsSSP3 protein with the native non‐necrosis‐inducing CcSSP37 protein. A total of five domains were chosen for analysis, including two domains that correspond to exon 1 and exon 2 of the untranslated mRNA sequence and three variable regions identified by pairwise alignment of the two proteins (Figure 5a).

As expected, infiltration of the recombinant SsSSP3 protein into petal tissue resulted in strong visual necrosis, whereas infiltration of recombinant CcSSP37 protein did not (Figure 5b). Of the eight chimeric proteins infiltrated into petal tissue, only CcSSP37 substituted with the exon 2 region of SsSSP3 (^Subs^SsSSP3 Ex2) was able to induce strong necrosis, suggesting that the region encoded by exon 1 is not responsible for necrosis‐inducing activity. These results align with the hypothesis that the N‐terminal region is predominantly a signal peptide.

Infiltration of the three chimeric proteins containing SsSSP3 variable regions (VR1, VR2, and VR3) in the CcSSP37 background did not result in strong necrosis. Quantitative necrosis area measurements showed that the chimeric ^Subs^SsSSP3 Ex2 protein was able to induce statistically similar levels of necrosis recorded for SsSSP3 and BcSSP2 (Figure 5c). All other chimeric proteins produced background levels of necrosis equal to or less than the native CcSSP37 protein. Furthermore, the chimeric ^Subs^SsSSP3 Ex2 protein produced symptoms as early as 2 hr post‐infiltration, which matched what was observed for SsSSP3 and BcSSP2 native recombinant proteins (Figure 5d). These data suggest that a specific conformation of the C‐terminal half of the SsSSP3 protein is essential for necrosis‐inducing ability, which may explain why C‐terminal tagged SsSSP3^T^and BcSSP2 ^T^ proteins had reduced necrosis‐inducing ability.

### 2.8 *bcssp2* knockout strains exhibit wild‐type in vitro growth and virulence

**Figure 6:**
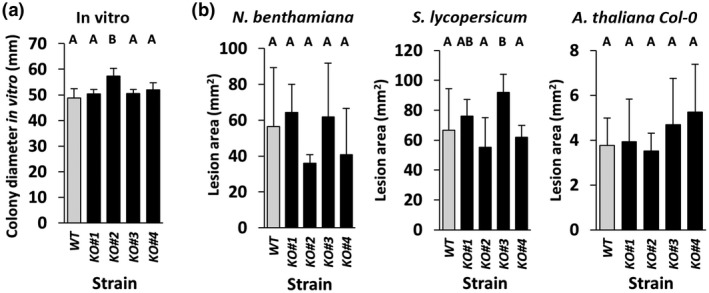
In vitro growth and in planta virulence assays of the four *Botrytis cinerea bcssp2* knockout (KO) strains. (a) In vitro growth of wild‐type (WT, grey bars) and four *bcssp2* knockout strains were (black bars) measured at 3 days post‐inoculation on malt extract agar (*n* = 10, Student's *t* test, *p *> .05, error bars = ±1 *SD*). (b) Comparative images and lesion area measurements of *bcssp2* KO and WT *B. cinerea* strains infecting leaves of *Nicotiana benthamiana* (*n* = 3), *Arabidopsis thaliana* (Col‐0) (*n* = 8) and *Solanum lycopersicum* (*n* = 6). Scale bar = 5 mm. Error bars = ±1 *SD*

To characterize the role of *BcSSP2* in *B. cinerea* virulence, four *bcssp2* knockout (KO) strains were created. *B. cinerea* was chosen for this experiment as it can be transformed readily. In vitro growth rate analysis on malt extract agar (MEA) showed that all four knockout strains were able to grow as well as the wild‐type (WT) strain (Figure 6a). Virulence assays on *N. benthamiana* (tobacco), *Solanum lycopersicum* (tomato), and *A. thaliana* Col‐0 leaves demonstrated that all four *bcssp2* knockout strains were able to develop lesions that were comparable in size to, or significantly larger (Tukey’s HSD *p* < .05) than, lesions induced by the WT strain (Figure 6b). These results indicate that *BcSSP2* is not essential for *B. cinerea* virulence.

## 3 DISCUSSION

Fungal secretome prediction is a popular strategy for identifying putative effectors that are required for fungal virulence or pathogenicity (Amselem *et al.*, 2011; Hacquard *et al.*, 2012; Morais do Amaral *et al.*, 2012). The objective of this study was to identify conserved proteinaceous fungal effectors by comparing the secretomes of three closely related, host‐diverse fungi: *C. camelliae*, *B. cinerea*, and *S. sclerotiorum*. The number of SSPs varied significantly between these three species due to the expansion of a family of SSPs in the host‐specialist *C. camelliae*. Recombinant protein assays indicated that several members of the CCL‐SSP family (BcSSP2 and SsSSP3) could induce host‐cell necrosis and that the C‐terminal half of SsSSP3 was essential for this necrosis‐inducing function. Knocking out *BcSSP2* in *B. cinerea* did not affect fungal growth in vitro or lesion size when compared to the wild‐type strain, suggesting that *BcSSP2* is not essential for full virulence.

The secretomes of *B. cinerea* and *S. sclerotiorum* have been predicted previously (Amselem *et al.*, 2011; Guyon *et al.*, 2014; Heard *et al.*, 2015). Guyon *et al. *(2014) predicted the secretome of *S. sclerotiorum* (*n* = 488) with a specific focus on secreted proteins expressed in planta (SPEPs). Heard *et al. *(2015) developed a rigorous prediction model to produce a “refined” secretome for both *B. cinerea* (*n* = 499) and *S. sclerotiorum* (*n* = 432). A total of 75% of the predicted SPEP sequences, 91% of the “refined” *B. cinerea* secretome, and 93% of the “refined” *S. sclerotiorum* secretome matched proteins within the *C. camelliae* secretome described here. In addition, BcSSP2 and SsSSP3 were predicted to be secreted fungal proteins in both studies.

Proteins that have previously been characterized as having a role in *S*. *sclerotiorum* virulence were successfully predicted in the secretomes described here, including SSITL (SS1G_14133) (Zhu *et al.*, 2013), Ss‐Caf1 (SS1G_02486) (Xiao *et al.*, 2014), and SsSSVP1 (SS1G_02068) (Lyu *et al.*, 2016). Furthermore, two *S*. *sclerotiorum* necrosis and ethylene‐like inducing proteins (NLPs), SsNEP1 (SS1G_03080) and SsNEP2 (SS1G_11912) (Dallal *et al.*, 2010), were also predicted. The *B*. *cinerea* NLP proteins BcNEP1 (BC1G_06310) and BcNEP2 (BC1G_10306) (Staats *et al.*, 2007) were successfully predicted in the *B. cinerea* secretome, as was the *B*. *cinerea* cerato‐platanin hypersensitive response (HR)‐inducing protein BcSpl1 (BC1G_02163) (Frías *et al.*, 2011). The prediction of these proteins confirms that the described secretome prediction pipeline successfully targets known virulence proteins of *S*. *sclerotiorum* and *B*. *cinerea*.

The CCL‐SSP family members share many characteristics with the NLP protein family. NLPs are virulence factors that are conserved in oomycetes, fungi, and bacteria (Qutob *et al.*, 2006). They universally share the sequence motif GHRHDWE, which is part of a 24‐amino acid motif that is thought to be recognized by nonspecific pathogen recognition receptors (Ottmann *et al.*, 2009; Oome *et al.*, 2014). These proteins are common in hemibiotrophic and necrotrophic microorganisms, and often exist as large gene families (Gijzen & Nurnberger, 2006). Although the CCL‐SSPs do not have the GHRHDWE motif, characteristics that are like the NLPs include the ability of the proteins to maintain function after heating (Oliveira *et al.*, 2012), their nonhost‐specific necrosis‐inducing function (BcSSP2 and SsSSP3), and their inclusion in the proteomes of multiple unrelated species. Like our observations of the *bcssp2* mutant strains, *B*. *cinerea bcnep1* and *bcnep2* NLP knockout strains have been shown to retain virulence (Arenas *et al.*, 2010). Future experiments should assess whether CCL‐SSP necrosis‐inducing function is dependent on secretion into the host apoplast (i.e., through the production of proteins that lack a signal peptide), a feature demonstrated previously for oomycete‐derived NLPs (Qutob *et al.*, 2006).

Nonhost‐specific SSPs have recently been reported for *Zymoseptoria tritici* (Kettles *et al.*, 2017). These SSPs were shown to induce nonhost cell necrosis when transiently expressed in *N*. *benthamiana*. Kettles *et al. *(2017) concluded that the nonhost necrosis phenotype was due to an interaction between pathogen recognition receptors (PRRs) that detect nonadapted pathogen proteins. The necrosis‐inducing phenotype observed for BcSSP2 and SsSSP3 may be a result of a nonspecific interaction with a PRR. The reduction of the necrosis‐inducing function observed for C‐terminus tagged recombinant BcSSP2^T^ and SsSSP3^T^ proteins suggests that slight changes in conformation affect the ability of these proteins to function in planta. We hypothesize that the necrosis‐inducing CCL‐SSPs described here are recognized by PRRs and act as microbe‐associated molecular patterns (MAMPs), comparable to what has been described for the nonhost‐specific *Z. tritici* SSPs and NLP superfamily members (Qutob *et al.*, 2006; Oome *et al.*, 2014; Kettles *et al.*, 2017).

To maintain virulence, fungal phytopathogens must constantly respond to selection pressure from the immune system of their host (Jones and Dangl, 2006). This evolutionary pattern is particularly true for host‐specific phytopathogens like *C. camelliae*. The birth‐and‐death evolution model has previously been used to describe the evolution of fungal effectors (Stergiopoulos *et al.*, 2012) whereby genes duplicate and diversify in response to host selection pressure (Nei and Rooney, 2005). Based on results presented here, the 73 *C. camelliae CCL‐SSPs* conform to the birth‐and‐death model. Evidence that *C. camelliae CCL‐SSPs* have increased their numbers through gene duplication includes their proximity to each other in the *C*. *camelliae* draft genome, their nucleotide conservation, and their conserved exon/intron structure. A similar scenario has been reported for SSPs in the biotrophic fungus *U. maydis*, where 12 genomic clusters of two to five SSP genes were discovered (Kämper *et al.*, 2006).

*C. camelliae* CCL‐SSPs appear to be diversifying and have a marked reduction in necrosis‐inducing ability compared to their single‐copy homologs in *B. cinerea* and *S. sclerotiorum*. Although the reduction of necrosis‐inducing ability in *C. camelliae* CCL‐SSPs associates with the evolution of *C. camelliae* host specificity, it remains unclear whether host selection pressure has contributed to the diversification of *C. camelliae CCL‐SSP* genes. Ma *et al.* (2017) demonstrated that *Phytophthora sojae* secretes decoy proteins during soybean infection that act to sequester plant inhibitor proteins in order for paralogous effector proteins to remain functional. An increased understanding of the endogenous function of *C. camelliae* CCL‐SSPs during early infection would elucidate whether a comparable decoy system has evolved in *C. camelliae* in response to host selection pressure.

Comparative analysis of the predicted *C. camelliae*, *B. cinerea*, and *S. sclerotiorum* secretomes identified proteins that conformed to the small secreted, cysteine‐rich profile of previously reported fungal effectors. A family of conserved SSPs was identified in *C. camelliae* (*n = *73), *B. cinerea* (*n = *1), and *S. sclerotiorum* (*n = *1). The lineage‐specific expansion of *C. camelliae CCL‐SSP* genes appears to have arisen through gene duplication and is possibly a result of host‐mediated adaptive evolution. Multiple *CCL‐SSP* homologs are conserved in other phytopathogenic fungi, suggesting that this family is universally important. Functional characterization of 12 CCL‐SSPs indicated that the *B. cinerea* and *S. sclerotiorum* recombinant protein homologs induce rapid host‐cell necrosis in a nonhost‐specific manner and that the C‐terminal region of the SsSSP3 protein is required for this phenotype. However, knocking out *BcSSP2* in *B. cinerea* suggested that BcSSP2 function is not required for full virulence. We speculate that the CCL‐SSP family may function by interacting with apoplast‐localized PRRs in a similar manner to other necrosis‐inducing fungal proteins.

## 4 EXPERIMENTAL PROCEDURES

### 4.1 Plant and fungal material

*Camellia* 'Nicky Crisp' (*Camellia japonica* × *Camellia pitardii* var*. pitardii*) shrubs were maintained in a glasshouse at ambient temperature. *Camellia* 'Nicky Crisp' petals were used for recombinant protein assays. *N. benthamiana*, *S. lycopersicum*, and *A. thaliana* ecotype Col‐0 were grown on seed raising mix (Daltons, NZ) at 22 °C, with a photon flux of 180 μmol⋅m^−2^⋅s^−1^, relative humidity of 75%, and a 10 hr:14 hr, light:dark photoperiod.

The *C. camelliae* isolate used in this study was collected from infected *Camellia* blooms from the Massey University Arboretum. Sclerotia were surface sterilized in 70% ethanol for 1 min and cultured on potato dextrose agar (PDA). A sample of the isolate that was used for genome sequencing was deposited in Landcare Research's International Collection of Microorganisms from Plants (ICMP), reference 19812. The *C. camelliae* draft genome sequence (accession PRJNA289037) (Denton‐Giles, 2014) and in planta RNA‐Seq data (accession SRS2024035) are available at GenBank.

*B. cinerea* strain B05.10 was maintained on MEA. To induce conidiation, cultures were incubated at room temperature in the dark for 3–5 days, placed under near‐UV for 24 hr, and then returned to the dark for 7 days. Conidia were harvested in 10 ml of milli‐Q water, filtered through sterile Miracloth, and pelleted by centrifugation. In vitro growth assays of wild‐type *B. cinerea* and *B. cinerea bcssp*2 knockout strains were performed by inoculating MEA plates with 5 mm plugs of mycelia and recording the colony diameter at 2, 3, and 4 days post‐inoculation.

### 4.2 Fungal secretome prediction, validation, annotation, and comparative analyses

*S. sclerotiorum* and *B. cinerea* protein sequences were downloaded from the fungal genome database hosted by the Broad Institute (Amselem *et al.*, 2011). The bioinformatic pipeline used for secretome prediction is outlined in Figure S1. Predicted secretome proteins were annotated using BLAST2GO v. 2 (Conesa and Götz, 2008). GenBank BLAST annotations and BLAST2GO enzyme codes were used to manually group the proteins into 32 common categories (Table S1). Proteins were conservatively annotated as SSPs if they were shorter than 200 amino acids in length and had ≥4% cysteine content (Kim *et al.*, 2016).

The three secretomes of *C. camelliae*, *B. cinerea*, and *S. sclerotiorum* were compared to each other using BLASTP (Altschul *et al.*, 1997). Alignments that produced an *E* value ≤10^−3^ and included at least 10% of the length of the query sequence were designated as matches. Unsuccessful matches were assigned an amino acid identity of 0%. Alignments of <20% amino acid identity were intrinsically not considered as matches by the BLASTP program and were given an amino acid identity of 0%. Each query sequence produced two amino acid identity scores (from the two species to which it was compared) which were graphed using the two‐dimensional scatterplot “smoothScatter” function in R v. 3.2.2 (*R* Foundation for Statistical Computing; http://www.r-project.org/). Scatterplots were independently generated for each of the three secretomes and were overlaid for comparison using the “Z project” function in ImageJ v. 1.48.

### 4.3 Phylogenetic tree analysis

The SSP family maximum likelihood phylogenetic tree was created in Geneious v. 6.1.5 using the CLUSTALW alignment tool and the PHYML plugin to build the tree from 1,000 bootstrap samples (Kearse *et al.*, 2012).

### 4.4 In silico characterization of SSPs

The 46 homologous *SSPs* identified in the *C. camelliae* secretome were used to screen the *C. camelliae* draft genome for additional family members using BLASTN. An alignment *E* value cut‐off of ≤10^−5^ identified 27 additional nonredundant *SSP* genes (Table S3). The full coding sequences of all 73 *C. camelliae SSP* genes were manually deduced from the draft genome (Table S3). Disulphide bond and connectivity predictions were performed using DISULPHIND v. 1.1 (Ceroni *et al.*, 2006). MEME v. 4.9 (Bailey *et al.*, 2006) was used to predict conserved motifs. All 75 SSPs were aligned to the GenBank nonredundant protein database using BLASTP. For each of the top alignments (*E* value cut‐off of ≤10^−3^) the number of nonredundant homologs was tallied (Table 1).

### 4.5 Gene expression analyses

*C. camelliae* transcripts were identified by aligning transcriptome data to the *C. camelliae* genome using BLASTN. To be certain that no plant transcripts were mistakenly assigned as fungal, all transcripts were required to pass the following three threshold parameters: (a) a *C. camelliae* draft genome alignment *E* value of ≤10^−100^; (b) ≥50‐fold increase in read counts between mock and infected reference transcriptomes; and (c) ≤20 read counts in the mock reference transcriptome. To quantify transcript abundance, RNA‐Seq reads were mapped to the reference *C. camelliae* transcriptome using BOWTIE2 v. 2.0 (Langmead and Salzberg, 2012). An in‐house Perl script was used to tally transcript‐aligned reads (https://mpcox.github.io/mapcount/).

For temporal RT‐qPCR analysis of *C. camelliae SSPs*, total RNA samples (*n* = 3) of infected *Camellia* 'Nicky Crisp' petal tissue (harvested at 0, 6, 12, 24, and 48 hr post‐inoculation) were converted to cDNA and amplified. Primer efficiency measurements and quantification cycle values were calculated using LINREGPCR v. 2012.1. Relative mRNA levels were determined by comparative quantification to the two fungal housekeeping genes *Nicotinamide adenine dinucleotide* (*NAD*) and *Tubulin* (*TUB*). Temporal *SSP* expression was normalized to 6 hr post‐inoculation levels.

### 4.6 Functional characterization of SSPs

BcSSP2 (BC1G_01444), SsSSP3 (SS1G_06068), 10 *C*. *camelliae* SSPs and eight SsSSP3/CcSSP37 chimeric proteins were synthesized by GenScript (Hong Kong, China; http://www.genscript.com). Codon optimization was performed based on *P. pastoris* codon usage. Synthesized coding sequences were cloned into the pPICZA expression vector. The EasySelect Pichia Expression Kit (ThermoFisher Scientific) was used for downstream recombinant protein expression.

For protein expression, single colony isolates of transformed *P. pastoris* strains were grown overnight in 5 ml of liquid buffered glycerol complex medium (BMGY) media (2% peptone, 1% yeast extract, 100 mM potassium phosphate (pH 6.0), 1.34% yeast nitrogen base, 4 × 10^−5^% biotin, and 1% glycerol). Cells were pelleted, resuspended in BMMY protein induction media (BMGY with 0.5% methanol instead of glycerol) and harvested after 48 hr. The supernatant was frozen in liquid N_2_ and stored at −80 °C. Prior to recombinant protein infiltration, frozen culture filtrate supernatant was defrosted and filter‐sterilized. To test the induction efficiency, aliquots of induced culture were collected at 0, 3, 6, 9, 12, 24, and 48 hr post‐induction and infiltrated into host tissue. Photographs of host tissue were taken at 0, 2, 6, 8, 12, and 24 hr post‐infiltration.

Western blotting of SSPs was performed by blotting protein from a Tris‐glycine SDS gel onto a polyvinylidene difluoride (PVDF) membrane at 0.15 constant amperage for 2 hr. The antimouse monoclonal (9E10) to c‐Myc antibody was diluted 1/1,000 and the antimouse horseradish peroxidase conjugate antibody was diluted 1/40,000. BM Chemiluminescence blotting substrate (Roche) was used to visualize the protein on X‐ray film. After exposure, PVDF membranes were loaded into a Fujitsu Intelligent Dark Box II equipped with a LAS‐1000 camera. Light emission was recorded by collecting cumulative images every 60 s for a total of 16 min. The final image was used for semiquantification of the tagged recombinant protein.

### 4.7 Generation of *B. cinerea bcssp2* knockout strains

Constructs for homologous recombination were generated using the OSCAR recombination vectors pOSCAR and pA‐hygr‐OSCAR (Paz *et al.*, 2011). A 1,002 bp fragment upstream of the *BcSSP2* gene and a 999 bp fragment downstream of the *BcSPP2* gene were amplified from *B. cinerea* genomic DNA using primer pairs BcUP_F/BcUP_R and BcDW_F/BcDW_R, respectively (Table S2). PCR fragments were gel purified using Zymoclean Gel DNA Recovery Kit (Zymo Research) and cloned into the pA‐Hyg‐OSCAR vector by incubating 20 ng of both 5′ and 3′ purified PCR flanking products, 60 ng pA‐Hyg‐OSCAR, 60 ng pOSCAR, and 1 µl BP clonase II enzyme mix (Invitrogen) at 25 °C for 16 hr. The reaction was terminated by adding 0.5 µl proteinase K (20 µg/µl) and incubating for 10 min at 37 °C. Transformants were validated by Sanger sequencing. The final construct contained the hygromycin B phosphotransferase (*hph*) resistance gene with the *Aspergillus nidulans trpC* promoter, spanned by the two *BcSSP2* flanking regions. The polyethylene glycol method previously described by Kars *et al. *(2005) was used to transform *B. cinerea* B05.10 protoplasts.

### 4.8 *B. cinerea* virulence assays

*Arabidopsis* virulence assays were performed on 4‐week‐old plants. Rosette leaves were inoculated with 5 µl of a 5 × 10^5^ suspension of wild‐type or mutant conidia in half‐strength potato dextrose broth. Lesion area was measured at 72 hr post‐inoculation using ImageJ v. 1.48. *N. benthamiana* and *S. lycopersicum* assays were performed on detached leaves of 4‐week‐old plants. Leaves were placed in a Petri dish on moist filter paper and inoculated with 5 µl of a 5 × 10^5^ suspension of wild‐type or *bcssp2* conidia. Lesion area was measured 48 hr post‐inoculation.

## AUTHORS CONTRIBUTIONS

M.D., H.M., R.E.B., M.P.C., C.M., and P.D. planned and designed the research. M.D., H.M., T.S., and Y.D. performed experiments. M.D., H.M., C.M., and P.D. analysed and interpreted the data. M.D., R.E.B., M.P.C., and P.D. wrote the manuscript.

## Supporting information


**FIGURE S1** The bioinformatic pipeline used for fungal secretome prediction

**FIGURE S2** Phylogenetic analysis of predicted small secreted proteins (SSPs) from *Ciborinia camelliae* (pink), *Botrytis cinerea* (blue) and *Sclerotinia sclerotiorum* (green). A well‐supported clade consisting of 46 *C. camelliae *SSPs and single homologs from *B. cinerea *(BC1T_01444), and *S. sclerotiorum* (SS1G_06068T0) is visible near the top of the tree. The phylogenetic tree was created from a CLUSTALW protein alignment of the SSP secretome data. The Geneious PHYML plugin was used to build the tree from 1,000 bootstrap samples

**FIGURE S3** Clustering of 73 homologous small secreted protein (*SSP*) gene loci within the *Ciborinia camelliae* draft genome. (a) A graphical representation of the proximal distance of homologous *SSP* genes from one another. (b) An example of a single 6 kb locus from scaffold 302 (37,309 bp). Independently identified SSP homologs from the secretome (pink) and preceding genome mining process (green) cluster within the same 6 kb locus

**FIGURE S4** A protein sequence alignment of all 113 CCL‐SSP family members, including homologs in other species. Cysteine residues located from cysteine positions 2 to 8 are highly conserved (≥95%)

**FIGURE S5** Functional assays using native recombinant small secreted proteins (SSPs). (a) *Camellia *'Nicky Crisp' petal tissue infiltrated with “empty vector” culture filtrate (left petal lobe) and eight individual recombinant proteins (right petal lobe) (*n* = 3). Photographs were taken at 0, 2, 8, and 24 hr post‐infiltration. (b) *Nicotiana benthamiana* leaf tissue infiltrated with culture filtrates containing “empty vector”, CcSSP93, BcSSP2, and SsSSP3 recombinant proteins at 48 hr post‐infiltration

**FIGURE S6** Functional protein assays using culture filtrate aliquots of BcSSP2 and SsSSP3 collected at different time points post‐induction with BMMY (0, 3, 6, 9, 12, 24, and 48 hr).* Camellia *'Nicky Crisp' petal tissue infiltrated with “empty vector” culture filtrate (left petal lobe) and BcSSP2 or SsSSP3 culture filtrates (right petal lobe) (*n* = 3). Photographs were taken at 0, 2, and 8 hr post‐infiltration

**TABLE S1** Secreted proteins of *Ciborinia camelliae*, *Botrytis cinerea* and *Sclerotinia sclerotiorum*

**TABLE S2** Primers used in this study

**TABLE S3** *Ciborinia camelliae*‐like small secreted proteins

## Data Availability

The data that support the findings of this study are openly available in GenBank at https://www.ncbi.nlm.nih.gov/genbank/, reference number PRJNA289037 (*C. camelliae* genome) and SRS2024035 (in planta RNA‐Seq data).
