# Supplementary material for: Conservation and expansion of a necrosis‐inducing small secreted protein family from host‐variable phytopathogens of the Sclerotiniaceae
Source: Mol Plant Pathol. 2020 Feb 15;21(4):512–26. doi: 10.1111/mpp.12913 (PMC7060139; doi:10.1111/mpp.12913)
Supplement: Supplementary file 1 — FIGURE S1 The bioinformatic pipeline used for fungal secretome prediction [file MPP-21-512-s001.docx]

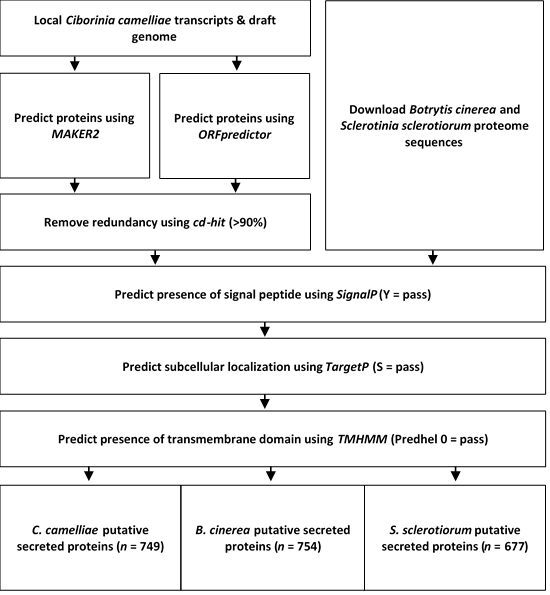


**Figure S1**

The bioinformatic pipeline used for fungal secretome prediction. For *Ciborinia camelliae,* the MAKER2 v2.26 software (Holt & Yandell, 2011) was used to predict protein-coding sequences from the *C. camelliae* draft genome and RNAseq data. The AUGUSTUS (Stanke *et al*., 2004) *ab-initio* component of MAKER2 was trained for *B.* *cinerea* gene prediction and applied to the *C. camelliae* genome. In parallel, *C. camelliae* proteins were predicted directly from RNAseq data using ORFPREDICTOR v1 (Min *et al*., 2005). The outputs of all three prediction strategies were combined and redundant sequences (i.e. ≥ 90% identity) were removed using cd-hit v4.6 (Fu *et al*., 2012). Predicted protein sequences were screened for signal peptides using SignalP v4.0 (Petersen *et al*., 2011), subcellular localization using TargetP v1.1 (Emanuelsson *et al*., 2000) and the presence of transmembrane domains using TMHMM v2.0 (Krogh *et al*., 2001). Only sequences that were ≥ 90 amino acids in length and had a methionine at their N-terminus were considered for TMHMM analysis.

**References**

**Emanuelsson, O., Nielsen, H., Brunak, S. and von Heijne, G.** (2000) Predicting subcellular localization of proteins based on their N-terminal amino acid sequence. *J. Mol. Biol.* **300**, 1005–1016.

**Fu, L., Niu, B., Zhu, Z., Wu, S. and Li, W.** (2012) CD-HIT: accelerated for clustering the next generation sequencing data. *Bioinform.* **28**, 3150–3152.

**Holt, C. and Yandell, M.** (2011) MAKER2: an annotation pipeline and genome-database management tool for second-generation genome projects. *BMC Bioinformatics*, **12**, 491.

**Krogh, A., Larsson, B., von Heijne, G. and Sonnhammer, E.L.L.** (2001) Predicting transmembrane protein topology with a hidden markov model: application to complete genomes. *J. Mol. Biol.* **305**, 567–580.

**Min, X.J., Butler, G., Storms, R. and Tsang, A.** (2005) OrfPredictor: predicting protein-coding regions in EST-derived sequences. *Nucleic Acids Res*. **33**, 677–680.

**Petersen, T.N., Brunak, S., von Heijne, G. and Nielsen, H.** (2011) SignalP 4.0: discriminating signal peptides from transmembrane regions. *Nat. Methods*, **8**, 785–786.

**Stanke, M., Steinkamp, R., Waack, S. and Morgenstern, B.** (2004) AUGUSTUS: a web server for gene finding in eukaryotes. *Nucleic Acids Res*, **32**, 309–312.
