# Supplementary material for: Conservation and expansion of a necrosis‐inducing small secreted protein family from host‐variable phytopathogens of the Sclerotiniaceae
Source: Mol Plant Pathol. 2020 Feb 15;21(4):512–26. doi: 10.1111/mpp.12913 (PMC7060139; doi:10.1111/mpp.12913)
Supplement: Supplementary file 2 — FIGURE S2 Phylogenetic analysis of predicted small secreted proteins (SSPs) from Ciborinia camelliae (pink), Botrytis cinerea (blue) and Sclerotinia sclerotiorum (green). A well‐supported clade consisting of 46 C. camelliae SSPs and single homologs from B. cinerea (BC1T_01444), and S. sclerotiorum (SS1G_06068T0) is visible near the top of the tree. The phylogenetic tree was created from a CLUSTALW protein alignment of the SSP secretome data. The Geneious PHYML plugin was used to build the tree from 1,000 bootstrap samples [file MPP-21-512-s002.docx]

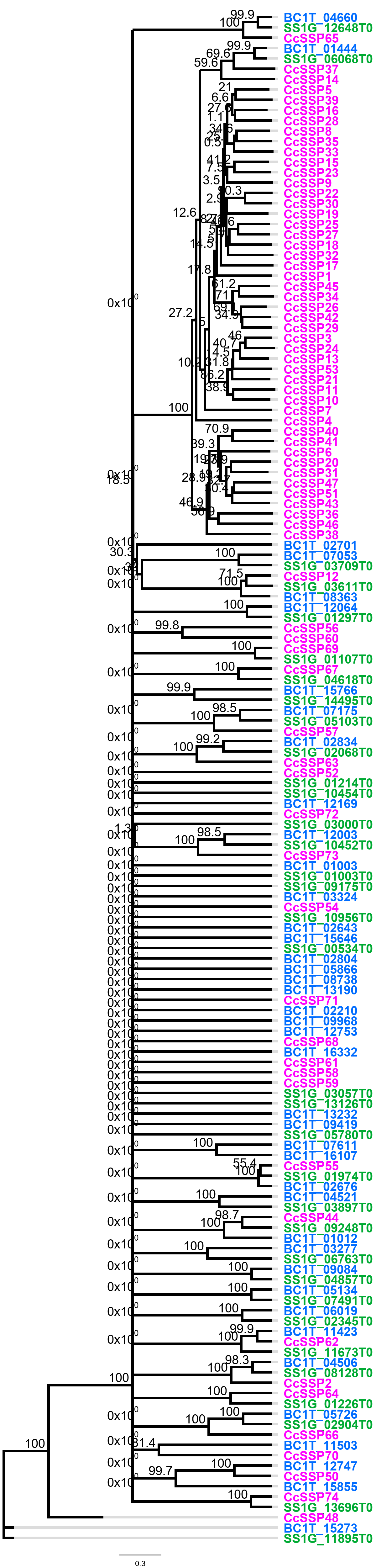


**Figure S2**

Phylogenetic analysis of predicted SSPs from *Ciborinia camelliae* (pink), *Botrytis cinerea* (blue) and *S. sclerotiorum* (green). A well supported clade consisting of 46 *C. camelliae* SSPs and single homologs from *B. cinerea* (BC1T_01444) and *S. sclerotiorum* (SS1G_06068T0) is visible near the top of the tree. The phylogenetic tree was created from a CLUSTALW protein alignment of the SSP secretome data. The Geneious™ PHYML plugin was used to build the tree from 1000 bootstrap samples.
