# Supplementary material for: Conservation and expansion of a necrosis‐inducing small secreted protein family from host‐variable phytopathogens of the Sclerotiniaceae
Source: Mol Plant Pathol. 2020 Feb 15;21(4):512–26. doi: 10.1111/mpp.12913 (PMC7060139; doi:10.1111/mpp.12913)
Supplement: Supplementary file 3 — FIGURE S3 Clustering of 73 homologous small secreted protein (SSP) gene loci within the Ciborinia camelliae draft genome. (a) A graphical representation of the proximal distance of homologous SSP genes from one another. (b) An example of a single 6 kb locus from scaffold 302 (37,309 bp). Independently identified SSP homologs from the secretome (pink) and preceding genome mining process (green) cluster within the same 6 kb locus [file MPP-21-512-s003.docx]

**A**

**B**


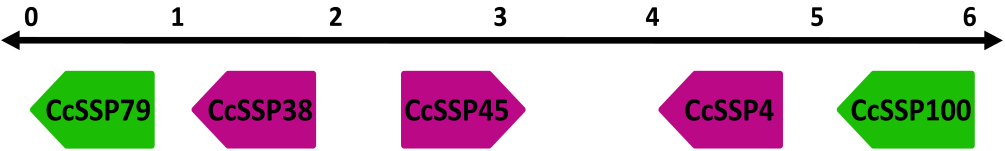


**Figure S3**

Clustering of 73 homologous small secreted protein (*SSP*) gene loci within the *C. camelliae* draft genome. (A) A graphical representation of the proximal distance of homologous *SSP* genes from one another. (B) An example of a single 6 kb locus from scaffold 302 (37309 bp). Independently identified SSP homologs from the secretome (pink) and subsequent genome mining process (green) cluster within the same 6 kb locus.
