# Supplementary material for: Conservation and expansion of a necrosis‐inducing small secreted protein family from host‐variable phytopathogens of the Sclerotiniaceae
Source: Mol Plant Pathol. 2020 Feb 15;21(4):512–26. doi: 10.1111/mpp.12913 (PMC7060139; doi:10.1111/mpp.12913)
Supplement: Supplementary file 4 — FIGURE S4 A protein sequence alignment of all 113 CCL‐SSP family members, including homologs in other species. Cysteine residues located from cysteine positions 2 to 8 are highly conserved (≥95%) [file MPP-21-512-s004.docx]

**
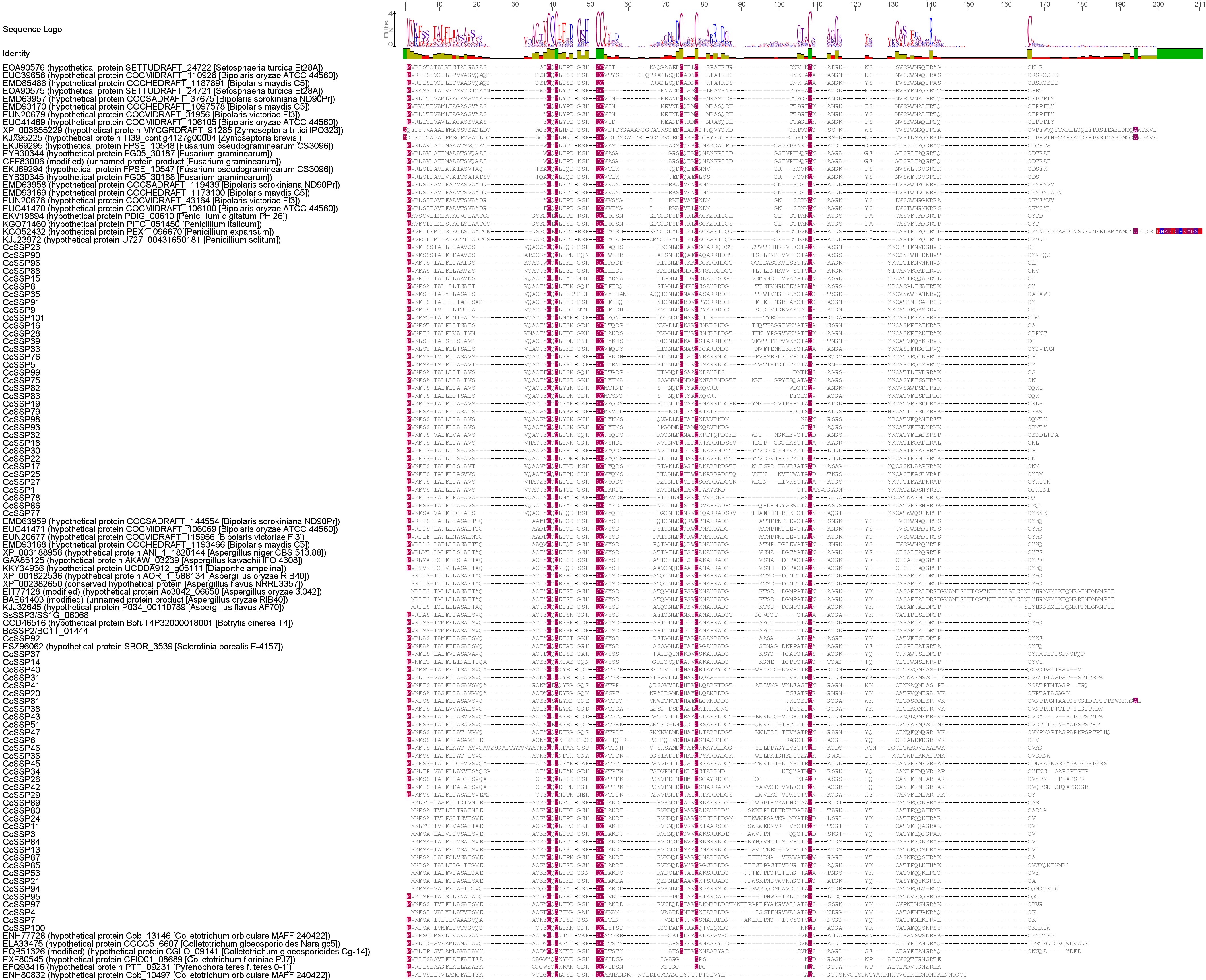
**

**Figure S4**

A protein sequence alignment of all 113 CCL-SSP family members, including homologs in other species. Cysteine residues located from cysteine position 2 to 8 are highly conserved (≥ 95%).
