# Supplementary material for: Conservation and expansion of a necrosis‐inducing small secreted protein family from host‐variable phytopathogens of the Sclerotiniaceae
Source: Mol Plant Pathol. 2020 Feb 15;21(4):512–26. doi: 10.1111/mpp.12913 (PMC7060139; doi:10.1111/mpp.12913)
Supplement: Supplementary file 5 — FIGURE S5 Functional assays using native recombinant small secreted proteins (SSPs). (a) Camellia 'Nicky Crisp' petal tissue infiltrated with “empty vector” culture filtrate (left petal lobe) and eight individual recombinant proteins (right petal lobe) (n = 3). Photographs were taken at 0, 2, 8, and 24 hr post‐infiltration. (b) Nicotiana benthamiana leaf tissue infiltrated with culture filtrates containing “empty vector”, CcSSP93, BcSSP2, and SsSSP3 recombinant proteins at 48 hr post‐infiltration [file MPP-21-512-s005.docx]

| **A** |  |  |  |  | **B** |  |  |
| --- | --- | --- | --- | --- | --- | --- | --- |
|  | **0 h** | **2 h** | **8 h** | **24 h** |  |  |  |
| **CcSSP33** | 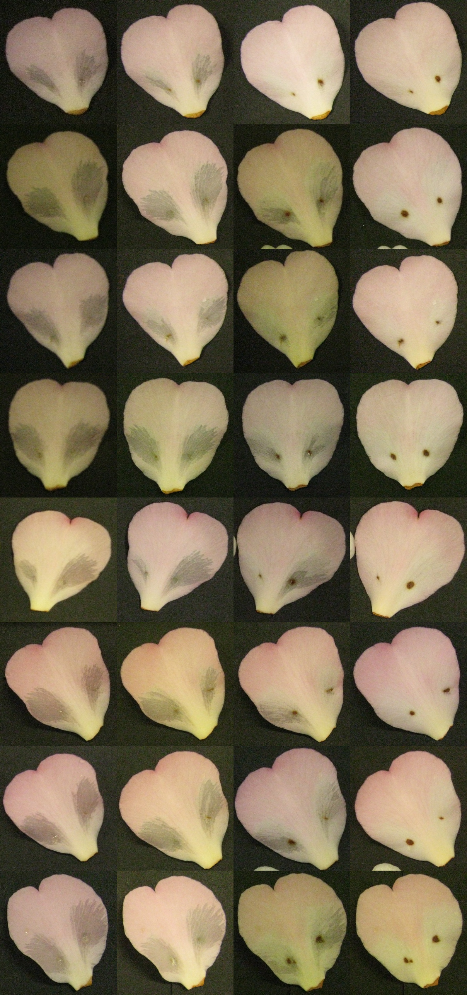 | | | |  | 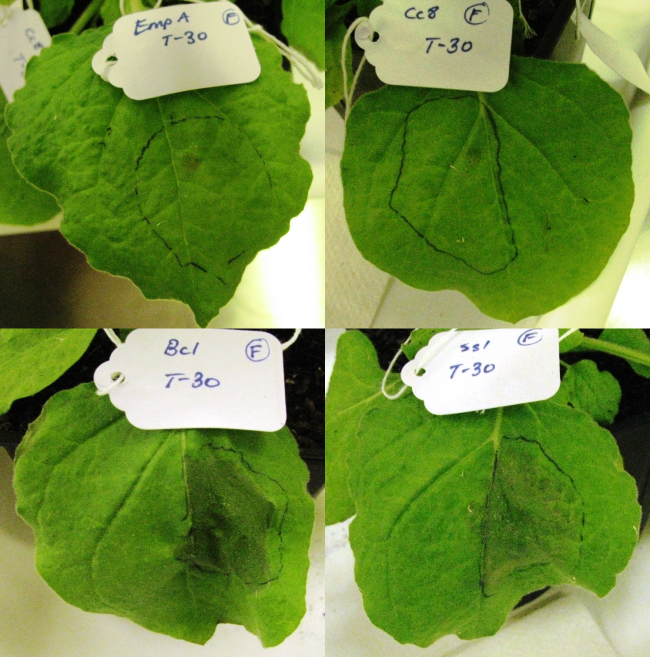  **SsSSP3**  **CcSSP93**  **Empty**  **BcSSP2** | |
| **CcSSP43** |  |  |  |  |  |  |  |
| **CcSSP31** |  |  |  |  |  |  |  |
| **CcSSP36** |  |  |  |  |  |  |  |
| **CcSSP41** |  |  |  |  |  |  |  |
| **CcSSP94** |  |  |  |  |  |  |  |
| **CcSSP93** |  |  |  |  |  |  |  |
| **CcSSP81** |  |  |  |  |  |  |  |

**Figure S5**

Functional assays using native recombinant small secreted proteins (SSPs). (A) *Camellia* ‘Nicky Crisp’ petal tissue infiltrated with “empty vector” culture filtrate (left petal lobe) and 8 individual recombinant proteins (right petal lobe) (*n* = 3). Photographs were taken at 0, 2, 8 and 24 h post-infiltration. (B) *Nicotiana benthamiana* leaf tissue infiltrated with culture filtrates containing “empty vector”, CcSSP93, BcSSP2 and SsSSP3 recombinant proteins at 48 h post-infiltration.
