# Supplementary material for: Conservation and expansion of a necrosis‐inducing small secreted protein family from host‐variable phytopathogens of the Sclerotiniaceae
Source: Mol Plant Pathol. 2020 Feb 15;21(4):512–26. doi: 10.1111/mpp.12913 (PMC7060139; doi:10.1111/mpp.12913)
Supplement: Supplementary file 6 — FIGURE S6 Functional protein assays using culture filtrate aliquots of BcSSP2 and SsSSP3 collected at different time points post‐induction with BMMY (0, 3, 6, 9, 12, 24, and 48 hr). Camellia 'Nicky Crisp' petal tissue infiltrated with “empty vector” culture filtrate (left petal lobe) and BcSSP2 or SsSSP3 culture filtrates (right petal lobe) (n = 3). Photographs were taken at 0, 2, and 8 hr post‐infiltration [file MPP-21-512-s006.docx]

|  | **BcSSP2** | | | **SsSSP3** | | |
| --- | --- | --- | --- | --- | --- | --- |
| **Hours post-induction** | **0 hpi** | **2 hpi** | **8 hpi** | **0 hpi** | **2 hpi** | **8 hpi** |
| **0** | 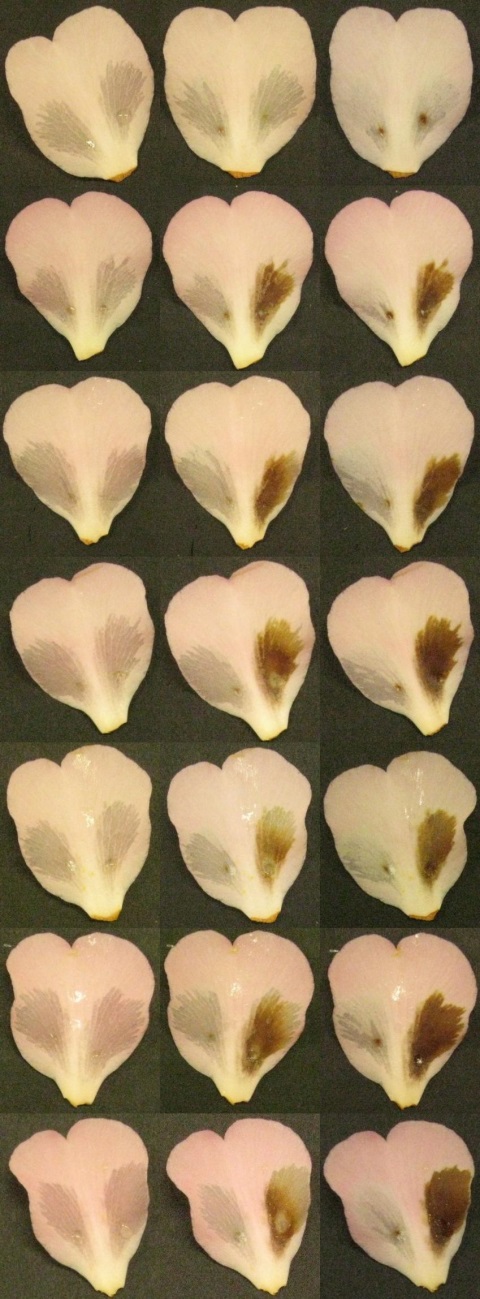 | | | 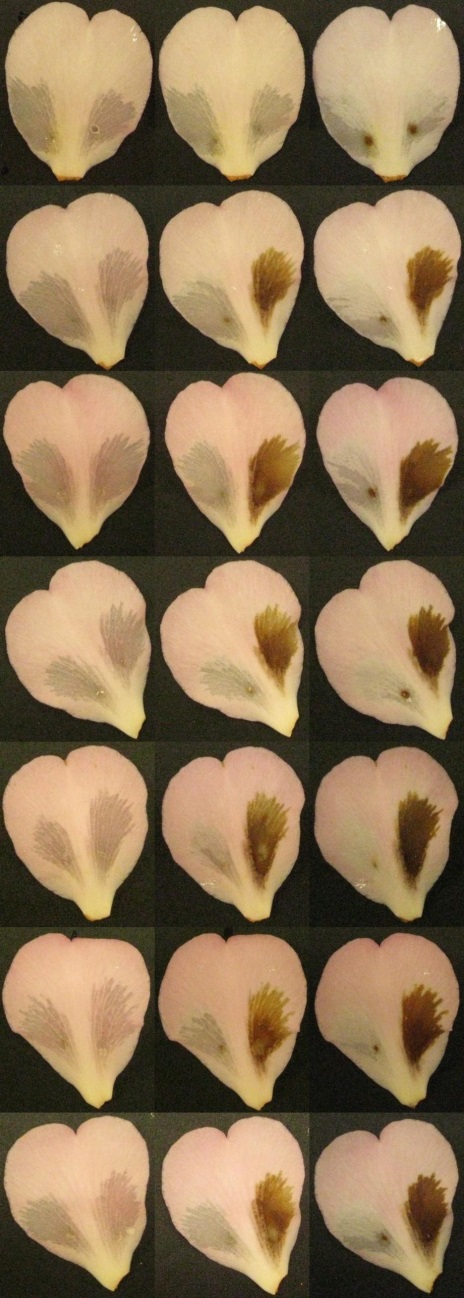 | | |
| **3** |  |  |  |  |  |  |
| **6** |  |  |  |  |  |  |
| **9** |  |  |  |  |  |  |
| **12** |  |  |  |  |  |  |
| **24** |  |  |  |  |  |  |
| **48** |  |  |  |  |  |  |

**Fig. S6** Functional protein assays using culture filtrate aliquots of BcSSP2 and SsSSP3 collected at different time-points post-induction with BMMY (0, 3, 6, 9, 12, 24 and 48 h). *Camellia* ‘Nicky Crisp’ petal tissue infiltrated with “empty vector” culture filtrate (left petal lobe) and BcSSP2 or SsSSP3 culture filtrates (right petal lobe) (*n* = 3). Photographs were taken at 0, 2 and 8 h post infiltration.
